# A daily positive work reflection intervention for psychological distress among Chinese nurses: a pilot randomized controlled trial

**DOI:** 10.3389/fpsyg.2025.1514612

**Published:** 2025-03-26

**Authors:** Li Zhang, Jian Xiao, Anao Zhang, Hui Zhang

**Affiliations:** ^1^Department of Cardiology, Taihe Hospital, Hubei University of Medicine, Shiyan, Hubei, China; ^2^Department of Otolaryngology, Sinopharm Dongfeng General Hospital, Hubei University of Medicine, Shiyan, China; ^3^School of Social Work, University of Michigan, Ann Arbor, MI, United States; ^4^School of Sociology, Huazhong University of Science and Technology, Wuhan, China

**Keywords:** daily positive work reflection intervention, nurse, psychological distress, randomized controlled trial, switching replication design

## Abstract

**Background:**

Chinese nurses experience high level of psychological distress, which negatively impacts nurses' mental health. A daily positive work reflection intervention is a widely disseminated employee management program, designed to improve employee wellbeing. The program has shown promising results in management, but has rarely been tested in healthcare settings, such as among nurses.

**Objective:**

The purpose of this study is to evaluate the efficacy of a daily positive work reflection intervention for psychological distress among Chinese nurses.

**Methods:**

This study used a switching replication design and randomly allocated 205 nurses to two groups (i.e., the experimental group and the waitlist control group) with three waves of measurement at pre-treatment, immediate post-treatment (T2), and immediate post-treatment (for the control group after intervention switch, T3) for changes in psychological distress.

**Results:**

In addition to significant within group improvements over time for both groups, OLS linear regression with Full Information Likelihood Estimation revealed a statistically significant between group treatment effects across outcome domains, including psychological distress, *b* = 22.60, *p* < 0.001, *g* = 11.34, somatic symptoms, *b* = 6.79, *p* < 0.001, *g* = 6.56, depressive symptoms, *b* = 8.15, *p* < 0.001, *g* = 8.19, and anxiety symptoms, *b* = 7.69, *p* < 0.001, *g* = 8.23.

**Conclusions:**

Results suggest that a daily positive work reflection intervention is a feasible and promising intervention for decreasing Chinese nurses' psychological distress. The study used a convenience sample which led to a concern of external generalizability, and the study had limited evaluation of long-term change.

## Introduction

Nurses are among the most vulnerable and stressful healthcare professions (Marey-Sarwan et al., 2022; Rafiei et al., 2024) and commonly face considerable levels of psychological distress such as stress, anxiety, and depression (Oldland et al., 2020; Vaismoradi et al., 2020). China has a large population and insufficient medical resources, which contributes to Chinese nurse experiencing higher work-related stress than their counterparts in developed countries (He et al., 2020). Previous studies suggested that Chinese nurses report high levels of care burden, long work shifts, shortage of staff and resources, physical and emotional pressure, dealing with death and dying, and even patient mistreatment or bullying (Xiao et al., 2022; Zhang et al., 2024), significantly impacting nurses' mental health and general wellbeing, including psychological distress (Rafiei et al., 2024). For example, a recent study involving 1,563 health professionals in China found that about half of the participants reported depressive and anxiety symptoms (Li et al., 2020).

Psychological distress is referred to as “the unique discomforting, emotional state [like depression or anxiety] experienced by an individual in response to a specific stressor or demand... to the person” (Ridner, 2004, p. 539). Previous empirical research has demonstrated that psychological distress among nurses would lead to negative outcomes across various domains, such as poor sleep quality (Olagunju et al., 2021), increased exhaustion (Anasori et al., 2019), burnout (Sexton and Adair, 2019), and somatic symptoms such as lower back pain (Farquharson et al., 2013) and an increased chance of being sick (Farquharson et al., 2013). Besides, nurses' psychological distress is predictive of their work engagement (Gomez-Salgado et al., 2021) and directly impacts the quality of care delivered to patients (Lu et al., 2017). Finally, the sequela of psychological distress among nurses is a high turnover rate (Farquharson et al., 2013; Hayes et al., 2012), reflecting the nursing workforce shortage both in China and internationally (Chan et al., 2013). Therefore, it is critical to address psychological distress among nurses (Hayes et al., 2010) and provide interventions to reduce their pyschological distress.

To date, existing efforts have tested the effectiveness of the intervention to reduce nurse psychological distress. A recent meta-analysis of nurse psychological distress intervention suggested that mindfulness intervention (Karo et al., 2024; Goldberg et al., 2018) and acceptance and commitment therapy interventions (Prudenzi et al., 2021) are effective in reducing nurse psychological distress, including stress and depression. These interventions are third-generation cognitive behavioral therapy modalities that mainly focus on improving cognition via awareness, attention, and self-regulation (Karo et al., 2024). Researchers also suggested that the interventions aimed at improving personal resources may also be effective in increasing wellbeing and reducing individual psychological distress (Clauss et al., 2018). One specific intervention is three good things intervention focuses on the general good things in their life (Guo et al., 2020) and positive work reflection, thinking and recalling about the positive work-related events after work (Meier et al., 2016). Besides, as nurses' psychological distress is positively related to employee daily work, positive work reflection developed by positive psychologists (Seligman et al., 2005) to develop the resources during the work, enhance employees' positive work experience, and trigger their positive emotions should be considered. Besides, rather than ask nurses to commit additional time and effort to learn a new skill, such as mindfulness, during a hectic work schedule, an ideal intervention should be person-centered, brief, and flexible. Positive work reflection intervention may ask individuals to think and recall positive work-related events after work, which is a brief and flexible intervention requiring only a few min to complete (Meier et al., 2016). Thus, the current study will examine the effectiveness of daily positive work reflection after work among Chinese nurses.

Theoretically, positive reflection helps employees recall positive experiences that may, in turn, trigger positive emotions (e.g., happiness), which can help employees build personal resources, such as hope and optimism (Clauss et al., 2018). Healthcare providers with higher levels of hope and optimism are more likely to experience lower psychological distress (Zhang et al., 2018). Besides, the daily positive work reflection intervention enables people to experience positive emotions, which replenish energy and help them to recover from exhaustion and fatigue (Oerlemans et al., 2014) and reduce psychological distress. Third, daily positive work reflection intervention can help nurses fight against the tendency to ruminate about negative events (Meier et al., 2016), which in turn leads to fewer experiences of negative affect and relieves psychological distress (Zhang et al., 2021). Fourth, by recalling/reflecting on positive events daily, nurses will have easier access to positive work events in their cognition, which facilitates savoring and capitalization (Gable et al., 2004) to reduce psychological distress.

Positive work reflection has been effectively used to enhance employees' wellbeing, foster personal resources (i.e., hope and optimism), and decrease exhaustion (i.e., emotional exhaustion and fatigue) in organization management (Bono et al., 2013; Clauss et al., 2018; Meier et al., 2016). For example, a daily positive work reflection intervention demonstrated improved results in promoting employee health, wellbeing (Bolier et al., 2013; Bono et al., 2013), positive affect (Meier et al., 2016; Rippstein-Leuenberger et al., 2017), and hope (Clauss et al., 2018), which is negatively related to psychological distress. Previous research also suggests that the daily positive work reflection intervention can reduce employee stress and fewer health complaints (Bono et al., 2013), negative emotions (Woodworth et al., 2016), emotional exhaustion (Clauss et al., 2018) and depression (Seligman et al., 2005; Sexton and Adair, 2019), which is positively related to psychological distress. Therefore, it is reasonable to expect a brief and low-cost strategy, such as a daily positive work reflection intervention, has the potential to offer low-resource, non-intrusive support to reduce psychological distress (Clauss et al., 2018).

**Figure 1:**
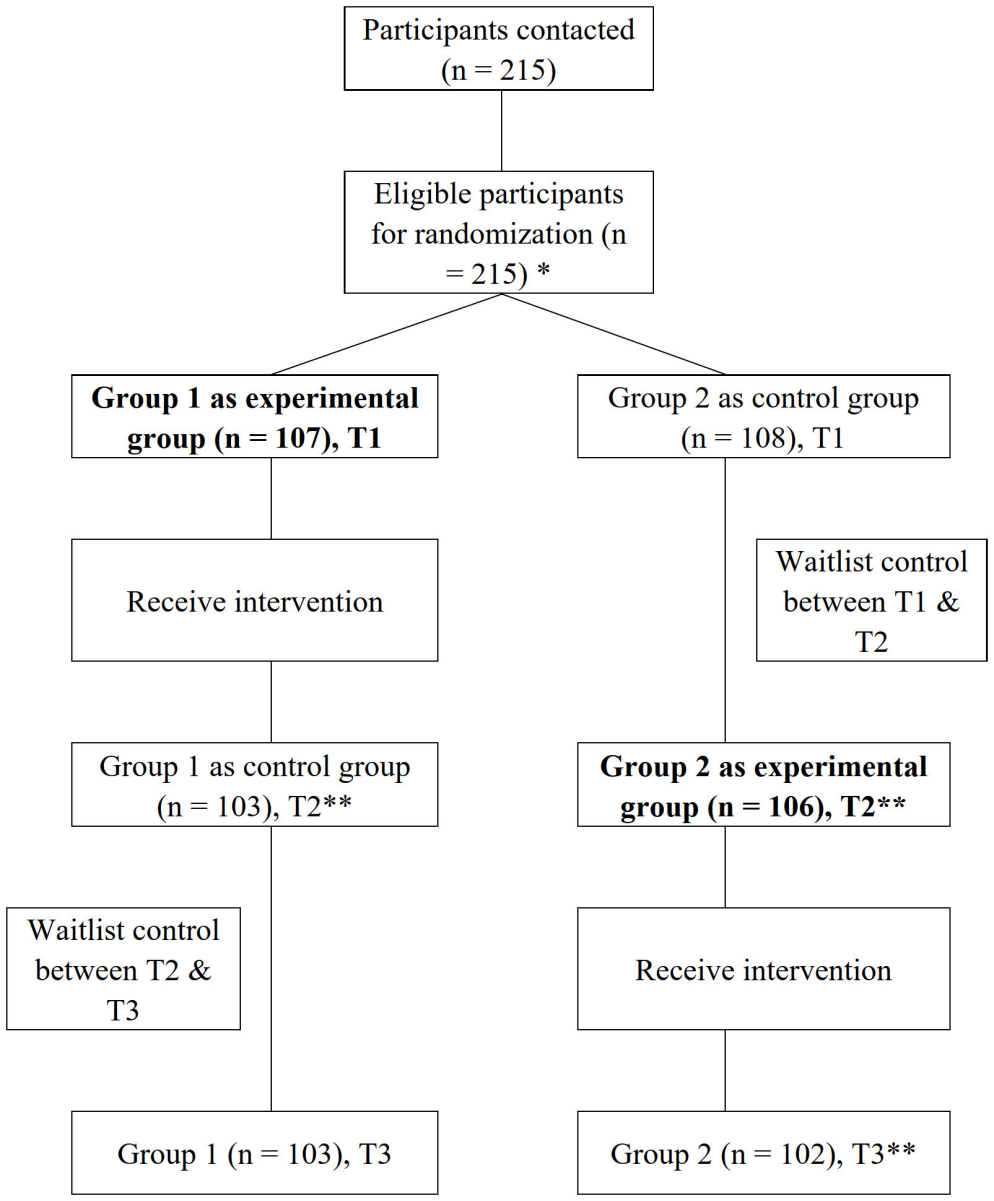
CONSORT flow diagram. ^*^Participants assigned to the Group 1 as experimental group at baseline (Time 1) received the intervention between Time 1 and Time 2, during which participants assigned to the Group 2 as waitlist control group at baseline (T1) served as waitlist control. After participants in the experimental group (Group 1) completed the intervention at time 2 (T2), those assigned to waitlist control at baseline (Group 2) switched as the experimental group and stared to receive intervention at T2 (2 weeks after T1). After the participants complete the intervention at Time 3 (T3) 2 weeks after T2, all participants completed the intervention. We measured participants' psychological distress at T1, T2, and T3. **Four dropped out of group 1 because the study was intervention too long (14 days) and cannot finish the intervention; 2 lost contact in group 2 while waiting in the waitlist control, and 4 dropped out of group 2 during the intervention.

Despite the above-mentioned supporting theoretical and empirical evidence, positive work reflection has been mainly used and evaluated in organizational management settings and rarely in healthcare settings. To provide a methodologically strong test of the effectiveness of a daily positive reflection intervention on nurses' psychological distress, we use an experimental switching replications design that incorporates two groups and three points of measurement unique to each group (Figure 1). This design is ideal for investigating both between- and within-person effects across time and ensures that both groups benefit from the intervention (Kiburz et al., 2017). Specifically, it can allow all participants to have the opportunity to receive intervention while simultaneously ensuring a methodologically rigorous design to evaluate the intervention's treatment effect. Thus, in this study, we tested the following hypotheses: (1) Participants in the experimental group receiving the daily positive work reflection intervention (between T1 and T2) will report lower levels of distress, somatization, depression, and anxiety than participants in the waitlist control group (waitlist control) at T2; (2) Participants in the waitlist control group receiving the daily positive work reflection intervention after work (between T2 and T3) will report no difference than those in the experimental group (waitlist control between T2 and T3) for distress, somatization, depression, and anxiety; and (3) Participants in both groups will report significant improvement between T1 and T3 for distress, somatization, depression, and anxiety.

## Methods

### Participant recruitment

Participants were nurses from a hospital in Hubei province, China, and were recruited from October 10th to December 14th 2020. We introduced the study to nurses in these hospitals with permission from hospital directors through in-person introductions and nurse-to-nurse introductions with the study's purpose, procedures, and inclusion/exclusion criteria. Nurses who were interested in the study completed an online questionnaire for eligibility screening. The study was approved by the Institutional Review Board.

#### Inclusion and exclusion criteria

To be eligible for participation, a participant needs to: (1) be a nurse at the time of enrollment; (2) meet the gender-specific baseline threshold (>10 for males and >13 for females) score of the Chinese version Brief Symptom Inventory (BSI-18)^1^ (Wang et al., 2013); (3) have a smartphone, laptop or desktop to receive and send messages in the during the study. We excluded participants who had a severe physical disease or cognitive impairment or were unwilling to provide consent. We also would exclude any participants who were currently not actively practicing nursing or were receiving ongoing technology-based mental health intervention.

### Procedure

This study used an experimental switching replication design to: (1) allow all participants have the opportunity to receive intervention while simultaneously (2) ensure a methodologically rigorous design to evaluate the intervention's treatment effect. The switching replications design includes two groups with three wave measurements at pre-treatment (T1), immediate post-treatment for the experimental group (T2), and immediate post-treatment for the control group (T3) (Figure 1). We used a computer-based random number generate to determine group assignment. Participants assigned to the Group 1 as experimental group at baseline (Time 1) received the intervention between Time 1 and Time 2, during which participants assigned to Group 2 as the waitlist control group at baseline (Time 1) served as waitlist control. After participants in the experimental group (Group 1) completed the intervention at Time 2, those assigned to waitlist control at baseline (Group 2) switched as the experimental group and started to receive intervention at Time 2 (2 weeks after T1). After the participants complete the intervention at Time 3 (T3) 2 weeks after T2, all participants completed the intervention. We measured participants' psychological distress at T1, T2, and T3. A statistician was blind to participant assignment. Given the nature of the intervention, we were unable to blind study participants, the psychologist, and the outcome assessor (research staff).

#### Treatment group

Participants in the treatment group received the daily positive reflection intervention after work. The intervention lasted 10 working days. Every day after work, the researchers would send the link to the participants to convey the intervention (5 p.m.). The intervention includes two steps. First, the participants were asked to think about the positive events during the work, which made them feel pleased and happy every working day. Second, the participants needed to write down three positive events during the work, such as the gratitude form the patients, the praise form leaders or coworkers or the accomplishment of a work and answer three questions to remember the detail of the three good things: “How did these positive events happen exactly?” (e.g., I got the gratitude and praise from the patients when I gave him some care to relieve his anxious. He said thanks so much for your care, I felt much better and I was very happy to talk with you); “Why did these events happen?” (e.g., I think the patient was so anxious about his disease and needed my care. I found his need and gave him some care proactively and professionally); and “How do you feel about the positive events?” (e.g., I think my work is meaningful and successful and I am very happy and proud that my work can help others actually). In order to promote the participation to intend the intervention, the researchers would send a reminder message to those who did not finish the intervention at 8 p.m. to encourage them to record the positive events happened during the work. The PhD-level psychologist was also available remotely throughout the study to address any immediate concerns or clinical deterioration.

#### Waitlist control group

Participants in the waitlist control group were informed to wait for 14 days before they start the intervention. During the 14-day period, if there was a significant deterioration in psychological distress, they can contact the research team for crisis intervention and obtain resources for mental health services.

### Measures

#### Psychological distress

We measured individual psychological distress using The Chinese version of the Brief Symptom Inventory (BSI-18) (Wang et al., 2013; Zhang et al., 2018), including three subscales (somatization, depression, and anxiety). The scale contains 18 questions, and some sample questions asked participants about short of breath, or feeling worthless, or feeling tense. Participants responded to a 5-point Likert scale from 0 (not at all) to 4 (extremely). The total score, in theory, ranges from 0 to 72, with a higher score indicating greater severity of psychological distress. Alphas across the three time periods ranged from 0.74 to 0.98.

We choose the measurement for the following reasons: (1) The BSI-18, as a shorter version of the 53-item measure of psychological distress used to screen for the most common psychiatric problems: somatization, depression, and anxiety, which improves the structural validity through an instrument with fewer domains (Derogatis, 2001); (2) Because of its simplicity, the BSI-18 is a useful measure to screen for psychiatric symptoms in a wide range of populations. Previous studies suggested that BSI-18 measurement has high reliability and validity (Zabora et al., 2001); (3) Study authors received permission to use this translated version of the Chinese version of BSI-18 for the current study. The Chinese version of BSI-18 has been systematically examined for its psychometric properties and found to have satisfactory validity and reliability when employed with a Chinese population (Wang et al., 2013).

### Data analyses

We needed 84 participants to achieve an 80% power for a moderate treatment effect (*g* = 0.4) using fixed-effect analysis of covariance (ANCOVA) for two groups at a critical alpha level of 0.05. While many nurses wanted to participate in the intervention, and we tried our best to provide intervention for the nurses, the current study enrolled 215 nurses in the intervention. Our final sample of participants completing the intervention (*n* = 205) passed the power analysis. Next, treatment effects were estimated using Ordinary Least Square (OLS) linear regression, with time being the focal predictor for within-group treatment effect and group assignment is being the focal predictor for between-group treatment effect for controlling age, gender, working years, educational level, and technical title. We also calculated within- and between-group small sample size corrected by Hedges' *g* to obtain treatment effect size, which is better for a small sample size (Hedges and Olkin, 1985). Finally, we conducted an intent-to-treat analysis using Full Information Maximum Likelihood (FIML) Estimation to address missing values, resulting in a final analytical sample of 205 participants.

## Results

### Recruitment, enrollment, and dropout

A total of 215 participants met the inclusion criteria and consented to complete all interventions. Eligible participants were randomly assigned to either the experimental group (*n* = 107) or the waitlist control group (*n* = 108). Four participants in the experimental group and six participants in the waitlist control group dropped out for reasons indicated in the CONSORT chart (Figure 1). A total of 205 nurses who completed the intervention sessions were included in the final data analysis, with four in the intervention group and six in the waitlist control group. Independent sample *t*-tests were conducted for comparing the continuous variables, and chi-squared tests were conducted for the categorical variables when comparing completers and non-completers. Results revealed that there was no significant difference between the completer (*n* = 103) and non-completer (*n* = 4) in assessment variables and the demographic characteristic of age, working years in the intervention group. In the control group comparison tests, there was no significant difference between the completer (*n* = 102) and non-completer (*n* = 6) in assessment variables and the demographic characteristic of age, working years. While as the number of categories in gender, educational level and technical level is too small, which is not suitable for chi-squared tests, we just show the number and percentage. More information about the comparisons of completers and non-completers can be found in Supplementary Tables 1, 2.

Participants had a mean age of 29.82 (*SD* = 6.00), most (*n* = 201, 93.49%) were female, most of them had university degree (*n* = 162, 75.34%) and approximately half of them were senior nurses (*n* = 105, 48.84%). On average, based on algorithm of the BSI-18 scoring manual (Recklitis et al., 2006), participants (from both groups) reported moderately severe psychological distress (mean = 33.03, *SD* = 5.13). More specifically, participants at baseline (T1) reported moderately high level of depressive symptoms (mean = 11.58, *SD* = 1.71) followed by moderate anxiety (mean = 10.37, *SD* = 1.71) and moderate somatic symptoms (mean = 10.37, *SD* = 2.07). No demographic or clinical characteristic information were statistically different between experimental group and waitlist control group, which supported the validity of randomization. Throughout the study, no participants reported concerns to self-harm or harming others. Table 1 presents detailed demographic and clinical characteristic information at baseline (Time 1).

**Table 1:** Group comparison of demographics and clinical characteristics at Time 1.

**Variable**	**Total**		**Group 1**		**Group 2**		**Between group difference**
	***M***	***SD***	***M***	***SD***	***M***	***SD***	**(*****t*****-test or** χ^2^)^*^
Age	29.82	6.00	29.43	5.92	30.20	6.09	−0.95
**Gender** ^**^							
Male	14		7		7		0.001
Female	201		100		101		
Working years	7.89	6.45	29.43	5.92	30.20	6.09	−1.08
**Educational level** ^**^							
Technical school degree	7		3		4		1.67
Junior college degree	42		18		25		
University degree	162		82		78		
≥ Master degree	3		2		1		
**Technical title** ^**^							
Nurse	65		37		28		3.45
Senior nurse	105		46		59		
Supervisor nurses	40		22		18		
Co-chief superintendent nurse	5		2		3		
Psychological distress	33.03	5.13	32.98	5.19	33.07	5.10	−0.13
Somatization	10.37	2.07	10.36	2.09	10.38	2.06	−0.05
Depression	11.58	1.71	11.55	1.73	11.61	1.70	−0.26
Anxiety	11.07	1.71	11.07	1.71	11.08	1.71	−0.08

* χ^2^ analysis was used to test the difference of gender, educational level, and technical title, t-test was used for others.

** For categorical variables, i.e., gender, education level, and technical title frequency was provided.

### Effects of the daily positive work reflection group intervention

**Figure 2:**
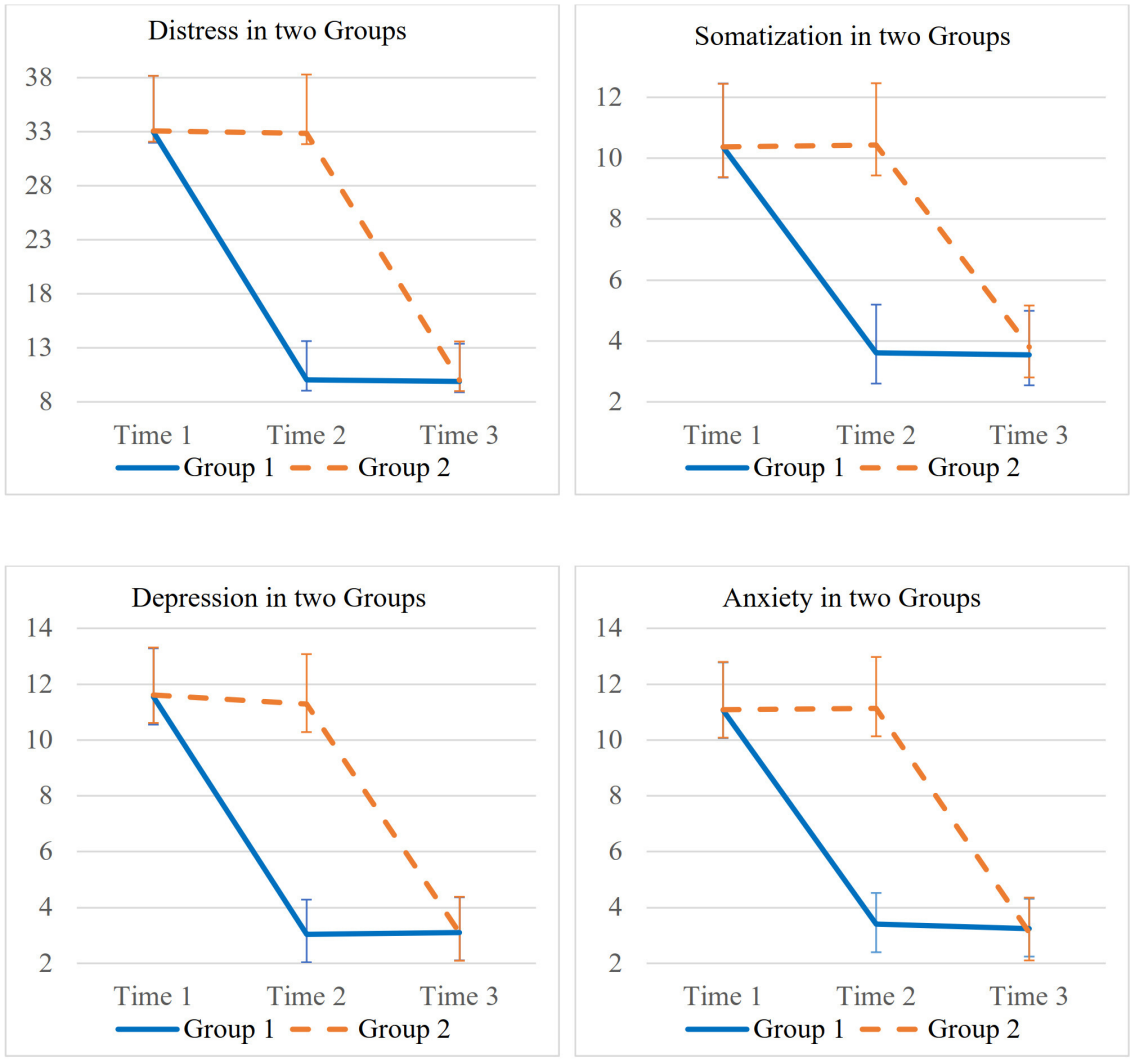
Line plots about Group 1 and Group 2.

The treatment effect was analyzed using between-group comparisons (Table 2). The between-group treatment effect at T2 revealed a statistically significant treatment effect of the daily positive work reflection group intervention for psychological distress compared to the waitlist control. Results of the analysis indicated that participants in the treatment group reported statistically significant better outcomes in psychological distress, *b* = 22.60, *p* < 0.001, *g* = 11.34, somatic symptoms, *b* = 6.79, *p* < 0.001, *g* = 6.56, depressive symptoms, *b* = 8.15, *p* < 0.001, *g* = 8.19, and anxiety symptoms, *b* = 7.69, *p* < 0.001, *g* = 8.23. Finally, after participants in the waitlist control group completed 10-working days intervention, the between-group difference was no longer statistically significant. Figure 2 presents the visual representation of participants' progress throughout the study.

**Table 2:** Between-group treatment effect at T2 and T3^*^.

	**Group 1 vs. Group 2 at T2**			**Group 1 vs. Group 2 at T3**		
	**Adjusted** ***b***	**SE**	**Hedges'** ***g***	**Adjusted** ***b***	**SE**	**Hedges'** ***g***
Distress	22.60^**^	0.28	11.34^**^	−0.03	0.23	0.02
Somatization	6.79^**^	0.14	6.56^**^	0.24	0.13	0.03
Depression	8.15^**^	0.14	8.19^**^	−0.08	0.13	0.09
Anxiety	7.69^**^	0.13	8.23^**^	−0.17	0.10	0.24

* Between-group treatment effect was evaluated controlling for T1 score, age, gender, educational background, and technological title; Full information maximum likelihood estimation was used to run the model to address missing value.

** *p* < 0.001.

Table 3 presents the within-group treatment difference and effect size. Participants in both groups reported statistically significant improvement before and after receiving the intervention. Upon completing the 10-working days intervention (between Time 1 and Time 2), experimental group participants reported significant decrease, total score in psychological distress, *b* = −22.90, *p* < 0.001, somatization, *b* = −6.75, *p* < 0.001, depressive symptoms, *b* = −8.50, *p* < 0.001, and anxiety symptoms, *b* = −7.66, *p* < 0.001. The within-group treatment effect remained after the participants finished the 10 working day's intervention for at least another 14 days (T3), evidenced by statistically significant improvement between T1 and T3 (results presented in Table 2). Within-group small sample size corrected Hedges' *g* (between T1 and T3) also revealed statistically significant large treatment effect size across all domains of outcomes, including psychological distress, *g* = 5.51, *p* < 0.001, somatization, *g* = 3.97, *p* < 0.001, depressive symptoms, *g* = 5.85, *p* < 0.001, and anxiety symptoms, *g* = 5.74, *p* < 0.001.

**Table 3:** Within-group treatment effect.

	**Group 1**				**Group 2**			
	**Time 1 M/SD**	**Time 2 M/SD**	**T1 to T2 Adjusted** b^*^	**Hedges'** ***g***	**Time 1 M/SD**	**Time 2 M/SD**	**T1 to T2 Adjusted** b^*^	**Hedges'** ***g***
Distress	32.98/5.19	10.04/3.59	−22.90^***^	–	33.07/5.10	32.85/5.44	−0.23	–
Somatization	10.36/2.09	3.60/1.59	−6.75^***^	–	10.38/2.06	10.43/2.03	0.05	–
Depression	11.55/1.73	3.04/1.24	−8.50^***^	–	11.61/1.70	11.28/1.79	−0.33	–
Anxiety	11.07/1.71	3.40/1.12	−7.66^***^	–	11.08/1.71	11.13/1.84	0.05	–
	**Time 2 M/SD**	**Time 3 M/SD**	**T2 to T3 Adjusted** b^*^	**Hedges'** ***g***	**Time 2 M/SD**	**Time 3 M/SD**	**T2 to T3 Adjusted** b^*^	**Hedges'** ***g***
Distress	10.04/3.59	9.89/3.49	−0.15	–	32.85/5.44	10.00/3.59	−22.85^***^	–
Somatization	3.60/1.59	3.54/1.45	−0.06	–	10.43/2.03	3.80/1.36	−6.63^***^	–
Depression	3.04/1.24	3.11/1.24	0.07	–	11.28/1.79	3.09/1.29	−8.19^***^	–
Anxiety	3.40/1.12	3.24/1.07	−0.16	–	11.13/1.84	3.10/1.25	−8.03^***^	–
	**Time 1 M/SD**	**Time 3 M/SD**	**T1 to T3 Adjusted** b^*^	**Hedges'** g^**^	**Time 1 M/SD**	**Time 3 M/SD**	**T1 to T3 Adjusted** b^*^	**Hedges'** g^**^
Distress	32.98/5.19	9.89/3.49	−11.53^***^	5.51^***^	33.07/5.10	10.00/3.59	−11.54^***^	5.27^***^
Somatization	10.36/2.09	3.54/1.45	−3.40^***^	3.97 ^***^	10.38/2.06	3.80/1.36	−3.29^***^	3.80^***^
Depression	11.55/1.73	3.11/1.24	−4.22^***^	5.85 ^***^	11.61/1.70	3.09/1.29	−4.26^***^	5.64^***^
Anxiety	11.07/1.71	3.24/1.07	−3.91^***^	5.74 ^***^	11.08/1.71	3.10/1.25	−3.99^***^	5.37^***^

* Within-group treatment effect was evaluated controlling for age, gender, educational background, and technological title; Full information maximum likelihood estimation was used to run the model to address missing value.

** Hedges' g _corrected_ results calculated the effect size of treatment within-group comparison from Time 1 to Time 3 using only complete cases.

*** *p* < 0.001.

Upon completing the 10-working days intervention (between Time 2 and Time 3), waitlist control group participants reported a significant decrease in total score in psychological distress, *b* = −22.85, *p* < 0.001, somatization, *b* = −6.63, *p* < 0.001, depressive symptoms, *b* = −8.19, *p* < 0.001, and anxiety symptoms, *b* = −8.03, *p* < 0.001. Within-group small sample size corrected Hedges' *g* also revealed statistically significant large treatment effect size across all domains of outcomes, including psychological distress, *g* = 5.27, *p* < 0.001, somatization, *g* = 3.80, *p* < 0.001, depressive symptoms, *g* = 5.64, *p* < 0.001, and anxiety symptoms, *g* = 5.37, *p* < 0.001.

## Discussion

With the global coronavirus pandemic further worsening the already high work stress among healthcare providers, nurses face significant challenges of psychological distress. While previous studies have suggested the effective intervention for psychological distress in field, to our knowledge, the current study among the first ones to investigate the effectiveness of a briefly and flexible positive work reflection intervention during 10 working days among Chinese nurses. The results showed that the positive work reflection intervention significantly reduced nurses' psychological distress. It was notable that both the between group and within group (i.e., pre and post) treatment effect was statistically significant. Besides, the statistically significant between- and within-group treatment effect, findings of this study also revealed statistically significant treatment effect for nurses' psychological distress indicating the intervention's clinical significance.

In addition to the treatment effect of positive reflection intervention on Chinese nurses' overall psychological distress, statistically significant treatment effects were observed across subdomains including depression, anxiety, and somatization. Although not definitive, robust findings across subdomains of psychological distress may suggest the trans diagnostic value of positive reflection on nurses' psychological distress, meaning that the intervention improves not just one but all aspects of psychological distress. Similar to previous work, positive reflection intervention may help reduce individual work exhaustion and depression (Clauss et al., 2018; Gold et al., 2023; Sexton and Adair, 2019), which contributes to the general psychological distress literature to show the value of positive work reflection in improving individual wellbeing and mental health.

As we conceptualized in our study, the benefits associated with positive work reflection intervention are considered to be a result of increased personal resources to cope with work strain (Sexton and Adair, 2019), including positive emotions (Meier et al., 2016; Rippstein-Leuenberger et al., 2017), hope and optimism (Clauss et al., 2018) and reduced rumination, which is negatively related to psychological distress. Specifically, the broaden-and-build theory (Fredrickson, 1998; Grant and Gino, 2010; Ouweneel et al., 2012) suggested that the experience of positive events builds personal resources, thus positive work reflection intervention helps employees recall positive experiences that may help them build personal resources, such as positive emotions (e.g., happiness), hope and optimism to recover from exhaustion and fatigue (Oerlemans et al., 2014) and reduce psychological distress. Second, positive reflection helps employees recall positive experiences and may also give rise to positive thoughts about the events of the ongoing working day, which in turn helps employees break the link between work stress (e.g., customer mistreatment) and psychological distress through rumination and build a replaced positive link between positive experience and positive outcome. Thus, employees may experience less negative affect and relieve psychological distress (Zhang et al., 2021). While the present study was not sufficiently powered to formally evaluate mediation, future studies would examine the mechanisms of positive affect, hope optimism in the perspective of resources and the mechanism of rumination in the perspective of cognition.

Our results also have implications for practice. Traditional interventions for individuals' psychological distress, such as mindfulness intervention, consist of eight 2 h weekly classes, an all-day training session, and 40 min of daily meditation homework (Perez-Blasco et al., 2013). As nurses face high work demands and less flexible time, the intervention costing lots of time may aggravate their pressure and they may not have enough time to accomplish the intervention. The positive work reflection intervention after work improves feasibility and accessibility while remaining effective and thus can be considered as an appealing alternative for clinical nurses with busy daily work. Finally, the positive work reflection intervention is not limited to the place and time and the results of the current study support an optimistic future for the application of the interventions within the larger population, particularly important for those who typically experience high workloads with few options for daily respite from work, such as the other high-stress professions. Thus, future research might use samples from other high-stress professions to compare and replicate the findings.

Given that the intervention lasts only 5–10 min and can be conducted at any time during the working day, it can be easily integrated into the daily work routine. For example, the hospital managers would ask the nurses to write down the positive events or share their experience with other nurses at the end of work in a team or department to expand the effect of the daily work reflection. Future studies may examine the effect of the intervention on the entire team instead of the single team members. Besides, nurse managers may also develop positive work reflection as an online self-help tool to progress long-term programs to enhance nurses' mental health, and future studies may examine the effect of an online intervention.

### Limitations and future research directions

This study has some limitations. First, the study used convenience snowball sampling with adults which led to a concern of external generalizability. Future studies should test the effectiveness of the intervention using larger samples and more diverse samples across the developmental spectrum (Xiang et al., 2020). Besides, we collected data from working nurses in China mostly are female, which is consistent with the gender distribution of nurses in China. While it may limit the generalizability of our findings to male nurses or other industries or countries. For example, women may be more sensitive to stress, and their psychological distress might be more affected. Thus, for a better understating of the effect of the positive work reflection on nurse psychological distress, future research could include a more diverse sample in terms of gender and professional backgrounds to ensure that the findings are more generalizable.

Second, we used waitlist control in our study which may introduce additional effects to the “true effects” of the intervention condition. For example, part of participants' improvement in the treatment condition may simply be due to attention or additional opportunities to socialize rather than the intervention. Given the nature of waitlist control as part of our design, we were unable to rule out these possibilities. However, considering both within and between-group effects were rather substantial, it is reasonable to expect the tested intervention to be effective. Besides, the participants in the waiting list control group may have high expectations for the intervention and be more engaged in the daily positive work reflection, which may enhance the effect of the intervention and future studies could be blind for the participants in the waiting list control group or tell them the intervention would begin at the time when they receive the intervention.

Third, previous studies suggested that workload fluctuations, recent exposure to traumatic events, or differences in hospital policies may significantly influence psychological distress (Rafiei et al., 2024), while considering to reduce the length of questionnaire and nurses time burden, the current study not measure these variables. Although the participants were randomized into either the intervention or the waiting list control group which can help minimize the impact of these variables, it is still a limitation and future studies should measure these variables and control the impact of these variables or examine the subgroup differences, providing a more comprehensive understanding of the intervention's effectiveness. In addition to the psychological distress, other outcome measures related to nurse-specific outcomes (e.g., burnout, job satisfaction) could provide a more holistic assessment of the intervention's impact.

Finally, although the majority of intervention studies measure the effects immediately or up to a few weeks after the intervention (Richardson and Rothstein, 2008), it would be worthwhile to implement intervention designs that exceed several months to capture the development of positive reflection and reduction of psychological distress over several months or years. Thus, the lack of evaluating long-term change is another limitation in the current study as we just monitor the practice of positive work reflection during the intervention. If the participants can make a practice of positive work reflection in their daily life after the intervention, the benefits of the intervention will be further improved. Thus, future studies should therefore measure the effects of positive work reflection on psychological distress after several months to assess the durability of the intervention's effects for the participants with continuous engagement in positive work reflection practice. This would also help determine if the benefits of the daily positive work reflection persist after the intervention ends. Besides, previous studies also suggested that the importance of individual-focused interventions in reducing psychological distress is complemented by organizational strategies that foster positive behaviors, such as organizational citizenship behavior (Mazzetti et al., 2022). Thus, future studies may examine the effect of the positive work reflection intervention on improving employees OCB.

## Conclusion

Results suggest that positive work reflection intervention can be a feasible and promising intervention for decreasing psychological distress among Chinese nurses. During the intervention, participants recalling the positive event during the work will have more personal resources to copy with work demand, and more positive affect to experience less psychological distress. The positive work reflection intervention as a low-cost strategy should be encouraged to be used in the nurse managements in more countries.

## Data Availability

The raw data supporting the conclusions of this article will be made available by the authors, without undue reservation.

## Footnotes

^1^To identify appropriate cutoff scores for the BSI-18, Zabora et al. (2001) found that the cases were separated by gender because the 25th percentile fell at a score of 10 for man and fell at 13 for women. To further substantiate the norms for the BSI-18, sensitivity and specificity were calculated to determine how well the BSI-18 identified positive cases using a score of 10 for men and 13 for women. Besides, previous studies also used the gender-specific thresholds in Chinees sample (Zhang et al., 2018, 2021). Thus, we also used the same gender-specific thresholds in Chinses nurses and encourage future studies to examine the gender-specific thresholds in the BSI-18 in Chinese sample.
