# Supplementary material for: A daily positive work reflection intervention for psychological distress among Chinese nurses: a pilot randomized controlled trial
Source: Front Psychol. 2025 Mar 26;16:1514612. doi: 10.3389/fpsyg.2025.1514612 (PMC11979135; doi:10.3389/fpsyg.2025.1514612)
Supplement: Supplementary file 1 [file Table_1.docx]

**Supplementary Materials**

**Table S1: Comparison of baseline demographic characteristics and baseline assessments between completers and non-completers in the intervention group (N=107)**

| **Variables** |  | **Intervention Group**  **Completer (*n*=103)** | | | |  | **Intervention Group**  **Non-completer (*n*=4)** | | | |  | ***p* value** | **Test-Statistics ^a^** |
| --- | --- | --- | --- | --- | --- | --- | --- | --- | --- | --- | --- | --- | --- |
|  |  | ***Mean*** | ***(SD)*** | ***n*** | **（%）** |  | ***Mean*** | ***(SD)*** | ***n*** | **（%）** |  |  |  |
| ^a^ Age |  | 29.45 | 5.99 |  |  |  | 29.00 | 4.55 |  |  |  | .88 | .15 |
| Working years |  | 7.46 | 6.49 |  |  |  | 6.00 | 5.29 |  |  |  | .66 | .45 |
| Psychological distress |  | 32.91 | 5.10 |  |  |  | 34.75 | 8.02 |  |  |  | .49 | -.67 |
| Somatization |  | 10.36 | 2.06 |  |  |  | 10.50 | 3.11 |  |  |  | .90 | -.13 |
| Depression |  | 11.50 | 1.67 |  |  |  | 12.75 | 2.63 |  |  |  | .16 | -1.42 |
| Anxiety |  | 11.05 | 1.67 |  |  |  | 11.50 | 2.36 |  |  |  | .61 | -.52 |
| **Gender** |  |  |  |  |  |  |  |  |  |  |  |  |  |
| female |  |  |  | 97 | (94.17%) |  |  |  | 3 | (75.00%) |  |  |  |
| male |  |  |  | 6 | (5.83%) |  |  |  | 1 | (25.00%) |  |  |  |
| **Educational level** |  |  |  |  |  |  |  |  |  |  |  |  |  |
| Technical School degree |  |  |  | 3 | (2.91%) |  |  |  | 0 | (/) |  |  |  |
| Junior college degree |  |  |  | 15 | (14.56%) |  |  |  | 3 | (75.00%) |  |  |  |
| University degree |  |  |  | 83 | (80.58%) |  |  |  | 1 | (25.00%) |  |  |  |
| ≥ Master degree |  |  |  | 2 | (1.94%) |  |  |  | 0 | (/) |  |  |  |
| **Technical title**** |  |  |  |  |  |  |  |  |  |  |  |  |  |
| Nurse |  |  |  | 35 | (33.98%) |  |  |  | 2 | (50.00%) |  |  |  |
| Senior nurse |  |  |  | 45 | (11.17%) |  |  |  | 1 | (25.00%) |  |  |  |
| Supervisor nurses |  |  |  | 21 | (20.39%) |  |  |  | 1 | (25.00%) |  |  |  |
| Co-chief superintendent nurse |  |  |  | 2 | (1.94%) |  |  |  | 0 | (/)2 |  |  |  |

^a^ Independent sample *t*-tests were conducted for the continuous variables.

**Table S2: Comparison of baseline demographic characteristics and baseline assessments between completers and non-completers in the waiting list control group (N=108)**

| **Variables** |  | **Intervention Group**  **Completer (*n*=102)** | | | |  | **Intervention Group**  **Non-completer (*n*=6)** | | | |  | ***p* value** | **Test-Statistics ^a^** |
| --- | --- | --- | --- | --- | --- | --- | --- | --- | --- | --- | --- | --- | --- |
|  |  | ***Mean*** | ***(SD)*** | ***n*** | **（%）** |  | ***Mean*** | ***(SD)*** | ***n*** | **（%）** |  |  |  |
| ^a^ Age |  | 30.26 | 6.09 |  |  |  | 29.17 | 6.56 |  |  |  | .67 | .43 |
| Working years |  | 8.44 | 6.53 |  |  |  | 7.00 | 5.66 |  |  |  | .60 | .53 |
| Psychological distress |  | 33.18 | 5.01 |  |  |  | 31.33 | 6.56 |  |  |  | .39 | -.86 |
| Somatization |  | 10.43 | 2.04 |  |  |  | 9.50 | 2.51 |  |  |  | .29 | 1.07 |
| Depression |  | 11.60 | 2.23 |  |  |  | 12.75 | 2.63 |  |  |  | .75 | -.33 |
| Anxiety |  | 11.15 | 1.67 |  |  |  | 10.00 | 2.28 |  |  |  | .11 | 1.61 |
| **Gender** |  |  |  |  |  |  |  |  |  |  |  |  |  |
| female |  |  |  | 96 | (94.18%) |  |  |  | 5 | (83.33%) |  |  |  |
| male |  |  |  | 6 | (5.88%) |  |  |  | 1 | (16.67%) |  |  |  |
| **Educational level** |  |  |  |  |  |  |  |  |  |  |  |  |  |
| Technical School degree |  |  |  | 4 | (3.92%) |  |  |  | 0 | (/) |  |  |  |
| Junior college degree |  |  |  | 23 | (22.55%) |  |  |  | 2 | (75.00%) |  |  |  |
| University degree |  |  |  | 74 | (72.55%) |  |  |  | 4 | (25.00%) |  |  |  |
| ≥ Master degree |  |  |  | 1 | (0.98%) |  |  |  | 0 | (/) |  |  |  |
| **Technical title**** |  |  |  |  |  |  |  |  |  |  |  |  |  |
| Nurse |  |  |  | 25 | (23.81%) |  |  |  | 3 | (50.00%) |  |  |  |
| Senior nurse |  |  |  | 56 | (54.90%) |  |  |  | 3 | (50.00%) |  |  |  |
| Supervisor nurses |  |  |  | 18 | (17.65%) |  |  |  | 0 |  |  |  |  |
| Co-chief superintendent nurse |  |  |  | 3 | (2.94%) |  |  |  | 0 | (/) |  |  |  |

^a^ Independent sample *t*-tests were conducted for the continuous variables.
